# Obstructive colorectal cancer presenting as constipation during pregnancy

**DOI:** 10.1590/0100-3984.2017.0207

**Published:** 2019

**Authors:** Tiago Kojun Tibana, Rômulo Florêncio Tristão Santos, Patrícia Costa de Oliveira Campos Marques, Edson Marchiori, Thiago Franchi Nunes

**Affiliations:** 1 Universidade Federal de Mato Grosso do Sul (UFMS), Campo Grande, MS, Brazil.; 2 Santa Casa de Campo Grande, Campo Grande, MS, Brazil.; 3 Universidade Federal do Rio de Janeiro (UFRJ), Rio de Janeiro, RJ, Brazil.

**Figure 1:**
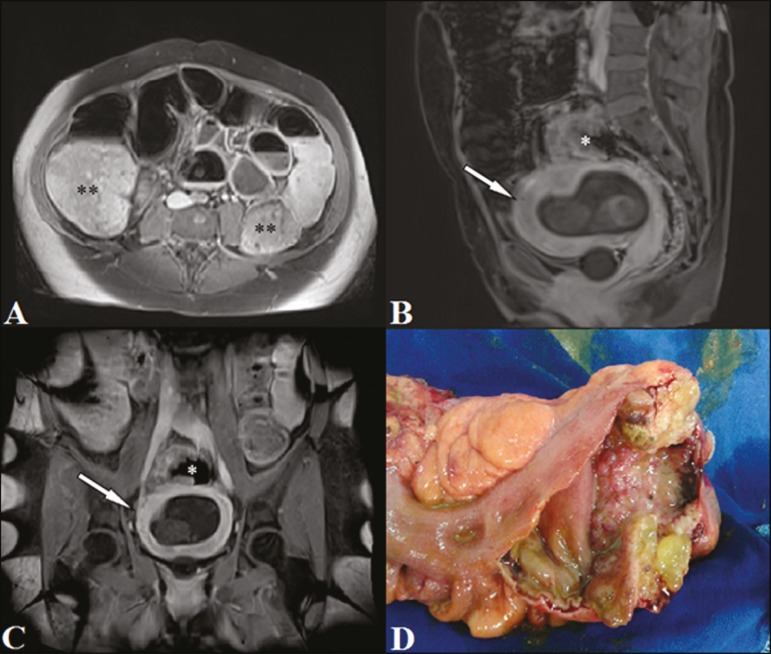
MRI of the abdomen in the axial plane (**A**), showing distention of the colon (asterisks). Images in the sagittal and coronal planes (**B** and **C**, respectively), showing an obstructive tumor in the lower rectum (asterisk) and a gravid uterus with the gestational sac (arrow). In **D**, surgical specimen showing an irregular, stenotic lesion.

Dear Editor,

A 36-year-old woman who was 16 weeks pregnant presented with chronic constipation that had worsened in the last 2 weeks, progressing to cessation of the elimination of gas and feces, together with abdominal distention and vomiting, as well as diffuse abdominal pain, predominantly in the left iliac fossa. A rectal enema provided no clinical improvement. The patient reported never having undergone surgery. Physical examination showed a distended abdomen and increased bowel sounds with a metallic tone. On deep palpation, there was pain, which was most severe in the left iliac fossa. There were no signs of peritonitis. Laboratory tests showed no significant alterations. Magnetic resonance imaging (MRI) of the pelvis showed diffuse distention of the colon (Figure 1A), with an expansile formation, at the rectosigmoid junction, characterized by irregular, concentric thickening, measuring 4 cm, and located approximately 20 cm from the anal canal (Figures 1B and 1C). No suspicious locoregional lymph nodes were observed. Conventional rectosigmoid resection and primary anastomosis were performed (Figure 1D). No macroscopic metastases were identified during the surgical procedure. A pathology study of the surgical specimen revealed an invasive, tubular, moderately differentiated, mucinous adenocarcinoma with lymphovascular invasion. Ultrasound in the immediate postoperative period showed a single fetus, with a heartbeat, and a normally implanted placenta. The evolution was satisfactory, and the patient was discharged on postoperative day 8.

The overall incidence of cancer in pregnant women ranges from 0.07% to 0.1%. Colorectal cancer during pregnancy is a rare entity, with an incidence of 0.002%^(^^1^^-^^3^^)^. There are a number of risk factors for colorectal cancer in pregnant women^(^^4^^)^: include advanced age; a personal or family history of adenomatous polyps; inflammatory bowel disease; a family history of colorectal cancer; a diet high in fat and animal protein; obesity; smoking; and alcohol consumption.

Mucinous adenocarcinoma is characterized by pools of extracellular mucin that compose more than 50% of the tumor volume. The mucinous component is one of the factors that influence patient survival. At any stage of differentiation, mucinous adenocarcinoma is considered a locally aggressive tumor with a poor prognosis^(^^5^^)^.

In pregnant women, acute abdominal symptoms often pose a diagnostic challenge. Although ultrasound is the first-line imaging method, additional tests are often required. With the development of faster sequencing techniques, MRI has come to provide important benefits, including multiplanar imaging and excellent soft tissue contrast, which, together with the fact that it does not involve the use of ionizing radiation, make it potentially more accurate than preoperative biopsy for the detection of mucinous adenocarcinoma^(^^6^^-^^10^^)^.

The treatment of colorectal cancer in pregnant women is complex and involves aspects such as gestational age of the fetus, tumor stage, and fertility preservation. During the first half of pregnancy, the treatment should be the same as that administered to a patient who is not pregnant. In the second half of pregnancy, the treatment should be postponed until the fetus is viable. After having given birth, the patient can undergo surgery. The main drugs used in adjuvant chemotherapy are considered safe for use in pregnant women from the second trimester onward. Radiotherapy is known to be beneficial in the preoperative and postoperative period of surgery for rectal tumors and can be indicated in special cases of tumors of the colon. However, it is contraindicated during pregnancy, and its effects on the fetus are unpredictable^(^^11^^)^.
